# Prolonged Lower Limb Dystonia and Dysphonia Following General Anesthesia in a Patient on Hydroxyzine

**DOI:** 10.7759/cureus.67263

**Published:** 2024-08-20

**Authors:** Tharaka Wijerathne

**Affiliations:** 1 Anaesthesia, The Rotherham Foundation Trust, Sheffield, GBR

**Keywords:** movement disorder, propofol, hydroxyzine, dysphonia, dystonia, general anaesthesia

## Abstract

We present a case of prolonged lower limb movement disorder following general anesthesia in a female patient in her early forties. She presented with vigorous, regular synchronous, rhythmic, and jerky movements during the immediate postoperative period lasting around forty minutes. Her past anesthetic history suggests varying degrees of postoperative movement disorders. Our patient was on long-term hydroxyzine for her skin condition. She had uneventful anesthetics before the prescription of hydroxyzine for her skin condition. All post-anesthetic dystonic events were reported while she was on hydroxyzine. Dystonic reactions during the perioperative period are rare and mostly occur during induction and emergence, which usually be transient. Our patient had prolonged lower limb dystonia resulting in severe muscular pain and lethargy for a few days. Further, she once developed transient aphasia and prolonged dysphonia following total intravenous anesthesia. This clinical finding could be a part of spasmodic laryngeal dystonia, which has not been reported previously. We correlate this rare postoperative dystonic reaction with propofol and possibly with the concurrent use of hydroxyzine.

As differential diagnosis can widely vary, the correlation of clinical findings with movement disorders is important for the diagnosis. Alterations of anesthetic techniques avoiding propofol and holding hydroxyzine are advisable in such rare clinical situations. Early diagnosis of perioperative movement disorders will prompt specific treatments, such as anticholinergic medications, for dystonia.

## Introduction

We report a case of prolonged dystonia on multiple occasions in a patient following general anesthesia. Movement disorders are rare during the perioperative period. However, transient movement disorders have been reported during induction and emergence from general anesthesia [1,2]. Severe and prolonged dystonic reactions following general anesthesia are rare, especially in patients with no other risk factors, and are frequently misinterpreted as convulsions.

Voice changes can be due to Laryngeal dystonia [3]; however, it has not been reported following general anesthesia. Our patient previously had aphasia and dysphonia following total intravenous anesthesia in the past which can be a presentation of laryngeal dystonia. Although hydroxyzine can rarely cause movement disorders [4], dystonia in patients on hydroxyzine has never previously been reported following general anesthesia. Even though not conclusive, we try to correlate dystonia as a possibility of concurrent use of hydroxyzine in our patient.

## Case presentation

A female patient in her early forties presented to us with severe sciatica pain for primary posterior fusion and decompression in the lumbar region. She had a medical history of chronic pain and skin psoriasis and had undergone a total of eight surgical procedures, including hand surgeries, arthroscopic shoulder surgeries, posterior decompression in the lumbar region, and partial gastrectomy as an emergency surgery in the past.

The patient experienced abnormal movements in her lower limbs during most of her postoperative periods. These were described as vigorous, painful, involuntary movements of the lower limbs lasting for a couple of hours. The use of midazolam and magnesium transiently reduced her signs and symptoms.

A year ago, she received total intravenous anesthetics for hand surgery, where she experienced aphasia for a couple of hours and dysphonia lasting for a couple of months. Dysphonia was explained as a stuttering and difficulty in controlling her voice with excessive loudness. She had no sore throat or structural anomaly on endoscopy. Her CT and MRI brain were essentially normal at that time and her symptoms as functional expressive dysphasia after referring to a neurologist.

She had no history of any movement disorders in the past except during the emergence of anesthetics. She had no family history of movement disorders or problems that could relate to anesthetics. She was on hydroxyzine for her skin condition as a long-term medication and took bisacodyl, co-codamol, lansoprazole, and methotrexate as regular medications.

On examination, she weighed 101 kg (BMI 38 kg/m²), was euthermic, and did not appear anxious. Her heart rate was 78 beats per minute, and her cardiovascular and respiratory systems were unremarkable. Apart from a foot drop on her left side, her neurological examination was normal. Her laboratory investigations, including electrolytes, were also normal.

Anesthesia was induced with 200 mg of propofol and 60 mg of rocuronium. During the 40 minutes of operative time, her vital parameters remained within normal ranges, and she was euthermic. Anesthesia was maintained with sevoflurane in oxygen and air. The patient received 4 mg of ondansetron, 6.6 mg of dexamethasone, a total of 15 mg of morphine, 1 g of paracetamol, 40 mg of parecoxib, 1 g of magnesium, and 1 mg of intrathecal morphine intraoperatively. After tracheal extubation, the patient was sent to the recovery area.

When she was fully awake, her legs began exhibiting regular, synchronous, rhythmic, and jerky movements. They were vigorous enough to cause her body to move or slide down the bed. The movements occurred in clusters of 4 to 5 jerks every 10 to 15 seconds. Her torso was also involved, with her abdominal and erector spinae muscles experiencing intermittent spasms. The patient’s lower limb muscles were spastic. She received 2 mg of midazolam, which transiently relieved her symptoms for about 2 to 3 minutes. The patient complained of severe pain in her lower limbs and was subsequently administered an additional 10 mg of morphine intravenously. No other abnormal movements or body positions were noted. She was fully conscious and cooperative throughout. Her heart rate increased to 140 beats per minute and remained above 120 beats per minute. The highest body temperature recorded was 36.8 °C.

After 40 minutes of dystonic movements, she was moved into an assisted sitting position with both legs hanging down, where complete cessation of her symptoms was noted. She experienced excessive sweating and muscle pain for a couple of hours, which were treated symptomatically.

The patient was discharged home on the second day, and no further abnormal movements were reported. She felt weak and fatigued for the following few days. Successive follow-up visits were carried out, and after multidisciplinary meetings with the anesthetic, acute medical, and specialist neurology teams, an anesthetic alert was raised and detailed communication was forwarded to her family physician. We suggested avoiding propofol and withholding hydroxyzine for at least three preoperative days in the future.

## Discussion

Dystonia is considered a movement disorder classically present with sustained muscle contractions and repetitive or patterned movements in varying body parts. It often produces a twisting posture and interestingly follows a task-specific movement, frequently called a *sensory trick.* Among the other hyperkinetic movement disorders like chorea, myoclonus, akathisia, tics, and tremors, our patient’s signs and symptoms tallied with dystonic movement disorder. She presents with this movement disorder only after anesthetics, therefore, it can be considered as a dystonic reaction to anesthetics or as a complex interaction of anesthetic with medication she is already on.

Sustained or patterned contractions of agonist and antagonistic muscles in limbs, trunk, and neck result in involuntary movements and twisting posture. She presented with lower limb dystonia; however, she suffered from dysphonia following anesthetics in the past, which can be a form of adductor laryngeal spasmodic dystonia resulting in voice tremors and affecting loudness of the voice. Further, spasticity in the neck and facial muscles could result in life-threatening airway emergencies with upper airway obstruction. To our knowledge, this is the first case report describing laryngeal dystonia following anesthetic.

Dystonic reactions during the perioperative period are rare and mostly occur during induction and emergence, which usually be transient [1,2]. The majority of the cases of perioperative movement disorders were correlated to propofol or antiemetic agents such as metoclopramide or ondansetron. In our case, ondansetron had been used without any ill effects previously. Propofol was used as the induction agent in all her previously reported dystonic events and, on one occasion, as part of total intravenous anesthesia, where severe lower limb dystonia and aphasia were reported.

A clear pattern of worsening severity and duration of the dystonic reaction after each anesthetic was noted in our case, and such a graded response following anesthesia has been described previously.

Even though the pathophysiology of dystonia is not clearly understood, several mechanisms have been described by various authors. The classic description is an imbalance between inhibitory dopaminergic and excitatory cholinergic systems in the basal ganglia. Recent research suggests that dystonia is a motor circuit disorder rather than a simple neurochemical imbalance confined to the basal ganglia. Further, studies involving tonic vibration reflex stimulus suggest that muscle spindle afferent pathways carry kinaesthetic inputs to the cerebellum [5]. Sensory inputs are directed to the basal ganglia and mainly to the striatum via the thalamus. These pathways could explain the sensory elements of dystonia.

**Figure 1:**
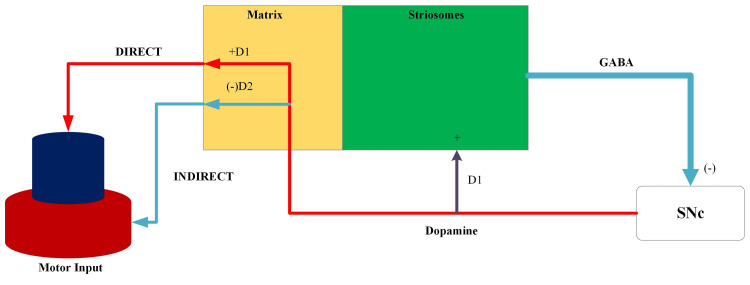
Three compartmental model of basal ganglia circuit with direct and indirect motor outputs. Source: [5]. GABA, gamma-aminobutyric acid; SNc, substantia nigra pars compacta

A compartment model in the striatum could explain some of the findings related to our patient’s presentation. Striosomes and matrices are considered neurochemically distinct compartments in the striatum (Figure 1). Of that, a much larger compartment, the matrix, comprises direct excitatory and indirect inhibitory neurons [5]. Excitatory outputs mediate via dopamine 1 (D1) receptors and indirect inhibitory outputs involve D2 receptors. Neurons in the ribosome get inputs from the limbic system. Cholinergic neurons influence the activation of striosomes. These ribosomal neurons have inhibitory projections into substantia nigra pars compacta (SNc) which act via gamma-aminobutyric acid (GABA) receptors. Outputs from SNc have feedback into striosome and matrix mediated via dopamine. It activates predominantly the direct pathway of matrix via D1 receptors giving rise to an excitatory motor response.

Various extrapyramidal side effects have been reported about the use of propofol. The mechanism of such movement disorders is largely unknown but commonly relates to an imbalance of cholinergic and dopaminergic effects in basal ganglia. Propofol is known to inhibit fatty amide hydrolase, which metabolizes the endocannabinoid anandamide (AEA). This leads to a significant increase in the whole brain content of AEA following propofol administration [6]. The endocannabinoid system has a fundamental role in the control of movements. Therefore, changes in the endocannabinoid system could partly explain the effects on movement disorders after propofol administration.

Hydroxyzine is a first-generation antihistamine that acts as an H1 receptor inverse agonist. It is commonly used in the treatment of itchiness, insomnia, motion sickness, and anxiety. Further, it has an antagonistic effect on D2 receptors. That could result in an inhibition of the indirect inhibitory pathway at the striatum, resulting predominance of excitatory direct motor output. There are cases of dystonia following hydroxyzine administration in the literature [4], and involuntary motor activities reported in hydroxyzine (ATARAXTM) product information. Studies have shown that long-term prescription of hydroxyzine can cause tardive dyskinesia [7].

Our patient was on hydroxyzine for her skin condition as a long-term medication. The patient had undergone a few general anesthetics without dystonic reactions before taking the treatment with hydroxyzine. However, since starting on hydroxyzine, she subsequently had dystonic reactions after each general anesthetic she had undergone. It is unclear how hydroxyzine interacts with general anesthetics, and we could not find previously published cases highlighting such interaction.

Acute dystonia is commonly treated with anticholinergic medications such as benztropine and procyclidine. Symptoms generally improve within 10 to 30 minutes. Intravenous benzodiazepines are effective in acute dystonic reactions.

## Conclusions

Movement disorders during the perioperative period are rare and transient. Atypical presentations of dystonic reactions can occur in the form of laryngeal dystonia and dysphonia. Even though postoperative dystonic reactions are commonly attributed to propofol and antiemetic medications, we conclude that they can be related to other dopamine antagonists like hydroxyzine. Therefore, detailed patient history, consideration of possible drug interactions, and correlation of clinical findings with movement disorders are all important for the diagnosis. Alterations of anesthetic techniques avoiding propofol and holding hydroxyzine are advisable in such rare clinical situations. Early diagnosis of perioperative movement disorders will prompt specific treatments, such as anticholinergic medications for acute postoperative dystonia.
